# GC-MS combined with multivariate analysis for the determination of the geographical origin of *Elsholtzia rugulosa* Hemsl. in Yunnan province

**DOI:** 10.1039/d2ra02876j

**Published:** 2022-08-02

**Authors:** Chaopei Zheng, Sifeng Yang, Dequan Huang, Deshou Mao, Jianhua Chen, Chengming Zhang, Weisong Kong, Xin Liu, Yong Xu, Yiqin Wu, Zhengfeng Li, Jin wang, Yanqing Ye

**Affiliations:** College of Chemical and Environment, Yunnan Minzu University Kunming 650500 China yey-qing@163.com; College of Chinese National Medicine, Yunnan Minzu University Kunming 650500 China; Research and Development Center, China Tobacco Yunnan Industrial Co., Ltd Kunming 650231 China wangjin@iccas.ac.cn

## Abstract

*Elsholtzia rugulosa* Hemsl., a Chinese herbal medicine, may have the potential to treat COVID-19. The geographical origin has a significant influence on the quality and application of *E. rugulosa.* In this paper, gas chromatography-mass spectrometry (GC-MS) combined with principal component analysis (PCA) and hierarchical cluster analysis (HCA) and other multivariate statistical analyses were performed for the identification of *E. rugulosa.* origins. The results showed that the volatile components of *E. rugulosa.* from different origins were significantly different. PCA and HCA can clearly distinguish the *E. rugulosa* of Lijiang and Fumin, and Dali and Yongsheng can be distinguished but with a certain overlap. The correlation of different components of was investigated by Pearson correlation. The results showed that *E. rugulosa.* characteristic component Elsholtzia ketone is regulated by terpenoid metabolism. The discriminant functions of different origins are constructed by Fisher stepwise discrimination, and its initial verification accuracy and leave-one-out cross-validation accuracy were 100% and 87.5%, respectively.

## Abstract 2

*Elsholtzia rugulosa* Hemsl., a Chinese herbal medicine, may have the potential to treat COVID-19, and its geographical origin has been successfully identified by GC-MS combined with multivariate analysis.

## Graphical abstract

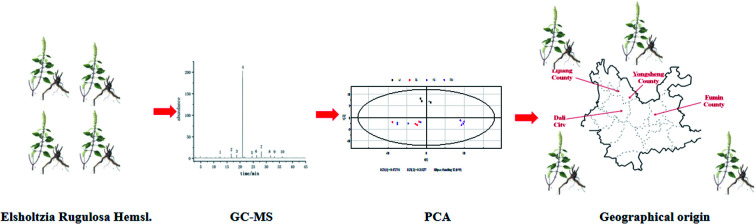

## 1. Introduction

At present, the Corona Virus Disease 2019 (COVID-19) is a global pandemic, and finding a suitable special medicine is an urgent problem to be solved. *Elsholtzia rugulosa* Hemsl. has been shown to have good antiviral, anti-influenza, and anti-inflammatory effects and may have the potential to treat and alleviate COVID-19.^1–6,9,16^*E. rugulosa* is a medicine and food homologous plant with a special fragrance.^1,2^ It is known as a local herbal tea, medicinal herb, and honey plant, mainly distributed in Southwest China, especially in Yunnan Province.^3–7^ In this region, the local ethnic minorities use it to treat colds, fever, flu, and diarrhea.^4–10^ In addition to the above-mentioned medicinal functions, recent studies have shown that many compounds isolated from *E. rugulosa* have anti-cancer and even relieve Alzheimer's symptoms.^4–18^ Through phytochemical methods, a variety of terpenoids, polyphenols, flavonoids, and other non-volatile components were identified, such as anti-inflammatory triterpene ursolic acid,^16,18^ antioxidant polyphenol rosmarinic acid,^26^ anti-influenza and anti-Alzheimer's flavonoid Luteolin,^11,12^*etc.* In the essential oil of *E. rugulosa*, β-dehydroelsholtzia ketone and Elsholtzia ketone were identified. In addition, various volatile medicinal components such as linalool,^27–29^ piperitone,^30,31^ isospathulenol,^31,32^ β-bourbonene,^34^ caryophyllene,^35^ α-humulene,^32–36^ spathulenol, artemisyl ketone,^34–36^*etc.* have been identified from the 21 species of *Elsholtzia*. As a herbal medicine, the safety, authenticity, and efficacy of *E. rugulosa* are all affected by the geographical origin. Therefore, it is necessary to trace the origin.

Origin traceability is an essential guarantee for the authenticity of the product, and the rights and interests of consumers. There are many research reports on the origin traceability of agricultural products such as wine, cheese, beef, rice, and wheat.^19–22^ These techniques of origin traceability include isotope analysis, multielement analysis, infrared spectroscopy, LC-MS, DNA method, *etc.* Gas chromatography-mass spectrometry (GC-MS) has good reproducibility, high sensitivity, and excellent separation and identification of volatile components.^23^ The GC-MS determination of volatile components can be used to make a preliminary evaluation of the medicinal efficacy of herbal medicines and further combined with chemometric methods can realize the traceability of its origin.

The primary aim of this research was to trace the origin of *E. rugulosa* from different origins. For the first time, GC-MS analysis technology, combined with multivariate statistical methods such as principal component analysis (PCA), hierarchical cluster analysis (HCA), and correlation analysis were used to analyze the volatilization of *E. rugulosa* from different origins. The *E. rugulosa* geographical origin discriminant functions were constructed by linear discriminant analysis. They were verified by initial validation and leave-one-out cross-validation (LOOCV).

## 2. Materials and methods

### 2.1 Reagents and chemicals

The HPLC-grade solvents methanol, chloroform, dichloromethane, ethyl acetate, and *n*-hexane were purchased from Beijing Chemical Reagent Company. Deuterated toluene (d8, >97%) as an internal standard was obtained from Sigma-Aldrich. Ultrapure water was prepared by a Milli-Q Water System (Millipore, Billerica, USA).

### 2.2 Sample collection and preparation

**Fig. 1:**
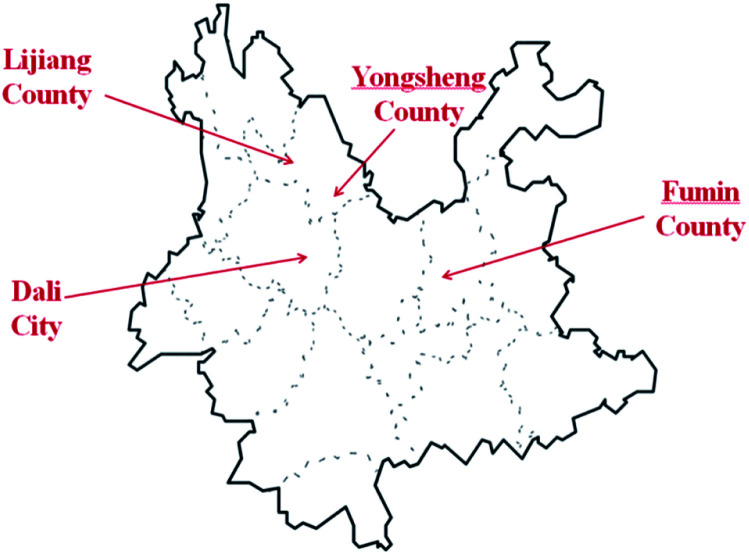
Geographical origins of the *E. rugulosa* samples from Yunnan Province: Lijiang (LJ), Yongsheng (YS), Fumin (FM), and Dali (DL).

The 16 *E. rugulosa* flower samples were collected from 4 different county of yunnan province in 2018–2019, including 4 from Fuming county (25° 22′N, 102° 34′E, 1685 m a.s.l), 4 from Dali county-level city (25° 42′N, 99° 93′E, 1976 m a.s.l), 4 from Yongsheng county (26° 32′N, 100° 66′E, 2140 m a.s.l), and 4 from Lijiang county (26° 86N, 100° 25′E, 2418 m a.s.l), with the geographic data from https://www.geodata.cn/. The locations of 4 sampling areas are marked on the Yunnan map shown in Fig. 1.

Samples were collected in the fall. All samples were dried in the sun and ground to obtain the powder.

### 2.3 GC-MS analysis

0.1 g ground sample was weighed in 10 mL centrifuge tube and 1.5 mL of 1 μg mL^−1^ deuterated toluene ethyl acetate solution was added. Then, the mixture was ultrasonically treated for 10 min, centrifuged at 4000 rpm for 8 min, filtered, 500 μL supernatant was transferred to a chromatography injection vial for the GC-MS test.

Gas chromatography (TRACE 1310, Thermo Scientific, USA) coupled with mass spectrometry (ISQ 7000, Thermo Scientific, USA) was employed to evaluate the volatile components in the sample. The column used was a DB-35 GC column (30 m × 0.25 mm × 0.25 μm) (Agilent, USA). The inlet temperature was 280 °C, and the helium gas flow rate through the column was 1 mL min^−1^. The injection volume was 1 mL, and the split ratio was 10 : 1. The initial oven temperature was 80 °C, heated to 275 °C at a rate of 60 °C min^−1^, then raised to 295 °C at a rate of 1 °C min^−1^, and held for 1 min. The transfer line and the ion source temperatures were 300 and 280 °C, respectively. The ionization mode was the electron impact at 70 eV. The solvent delay was 2 min.

### 2.4 Data pre-treatment and statistical analysis

The data pre-treatment is preprocessed as follows:^31,35,36^

#### 2.4.1 Filter

Filter a single peak to remove less than 50% of the peaks in a single group.

#### 2.4.2 Complementary value

Simulate the missing value in the original data, take half of the minimum value to fill.

#### 2.4.3 Normalization

Quantification using an internal standard method. The content of each volatile component is calculated as follows.
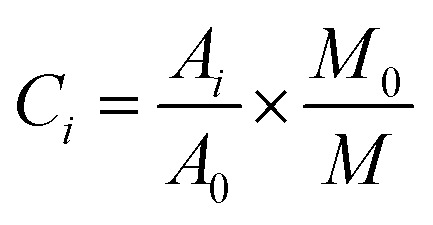


Among them: *C*_*i*_ represents the content of the measured odor components; *A*_*i*_ is the peak area of each compound; *A*_0_ represents the peak area of the internal standard substance; *M*_0_ represents the mass of the internal standard substance; *M* is the mass of the sample taken. The data is then used for statistical analysis.

Analysis of variance (ANOVA) was carried out for each element using SPSS 22.0 software (IBM, US). The significance level was *P* < 0.05. And the SPSS 22.0 is also used to correlation analysis, Fisher Discriminant analysis, initial validation, LOOCV. PCA multivariate analysis was performed by SIMCA-P 11 (Sartorius Stedim, Germany). Hierarchical cluster analysis (HCA), Heatmap and other plots were used OriginPro 2017 (OriginLab, US).

## 3. Results and discussion

### 3.1 GC/MS analysis

**Fig. 2:**
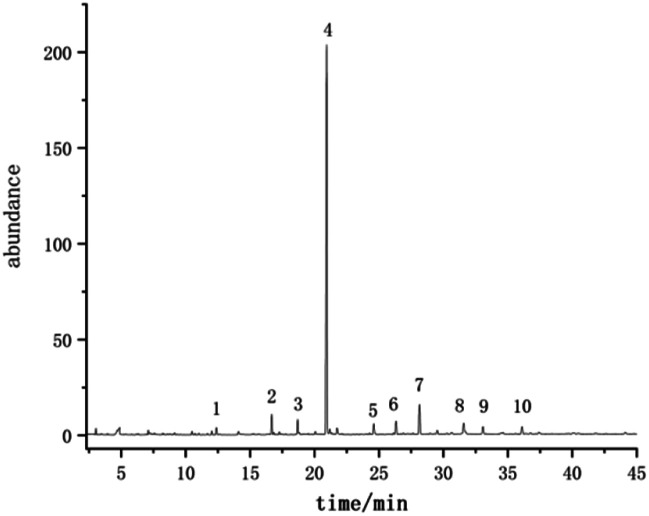
The total ion chromatogram of *E. rugulosa* (Lijiang sample; (1) artemisyl ketone, (2) 4-acetyl-1-carbamoyl-5-methylpyrazole, (3) methyl 5-hydroxyiminopentanoate, (4) β-dehydroelsholtziketone, (5) (−)-humuleneepoxideII, (6) isospathulenol, (7) diepicedrene-1-oxide, (8) hexadecanoic acid, (9) *N*-methyl-3-azabicyclo[3.1.0]hex-1-ylamine, (10) ethyl linoleate).

The volatile compounds of *E. rugulosa* flowers from 4 different regions were evaluated by GC-MS. The total ion chromatogram is shown in Fig. 2. Volatile compounds were identified by the comparison of the mass spectra with the commercial mass spectra libraries (NIST27 and WILEY7). Nearly 80 volatile components were separated and determined, and the data were analyzed by ANOVA, and the results are shown in Table 1. Among them, Elsholtzia ketone, linalool, artemisyl ketone, humulenol, isospathulenol, andrographolide, and many chemical components isolated from *E. rugulosa* have bio-medical activities such as anti-cancer, anti-inflammatory, anti-oxidation, anti-malarial, and anti-influenza virus.^5–18^ These compounds are also the material basis of the medicinal effect of *E. rugulosa*. It can be seen from Table 1 that under the significance level *P* < 0.05, the differential volatile components of *E. rugulosa* in the four origins reach more than 50, indicating that the origin has a great influence on the volatile components of *E. rugulosa*. In addition to *E. rugulosa.* characteristic components Elsholtzia ketone and β-Dehydroelsholtzia ketone, a variety of biologically active or medicinal components are also identified, such as artemisyl ketone, linalool, piperitone, isospathulenol, β-bourbonene, dimethyl-heptadienone, caryophyllene, α-humulene, spathulenol, (1R,7S,E)-7-isopropyl-4,10-dimethylenecyclodec-5-enol, andrographolide, thunbergol, squalene, β-sitosterol, and so on.

**Table 1:** GC-MS and its ANOVA data of *E. rugulosa* samples from 4 origins in Yunnan Province^a^^,^^b^

RT (min)	Compounds	Lijiang (μg g^−1^)	Dali (μg g^−1^)	Yongsheng (μg g^−1^)	Fumin (μg g^−1^)	*F*	*P*-Value
3.23	2,4-Dimethyl-1-heptene	0.56 ± 0.13	0.25 ± 0.25	0.21 ± 0.06	0.38 ± 0.25	2.49	0.11
3.63	3-Methyl-butanoic acid	2.34 ± 0.23	0.3 ± 0.3	0.28 ± 0.03	5.23 ± 0.35	175.99	0.00
4.96	3-Methyl-2-butenoic acid	11.18 ± 2.28	13.32 ± 2.92	6.17 ± 5.06	7.03 ± 0.91	1.77	0.21
5.43	Furan	1 ± 0.07	1.03 ± 0.16	0.42 ± 0.43	0.86 ± 0.11	1.74	0.21
6.09	3-Methyl-2(5*H*)-furanone	3.49 ± 0.37	3.97 ± 1.05	1.8 ± 1.56	1.85 ± 0.13	5.93	0.01
7.14	2-Methylpropyl 3-methylbutanoate	—	—	—	1.85 ± 0.06	2478.50	0.00
8.8	*trans*-Linalool oxide (furanoid)	1.65 ± 0.12	2.35 ± 0.56	1.01 ± 0.96	2.13 ± 0.08	3.22	0.06
8.8	(2R,5S)-linalool oxide(5) acetate	1.87 ± 0.12	1.74 ± 1.15	1 ± 0.67	2.67 ± 0.12	1.95	0.17
9.65	l-Linalool	0.81 ± 0.07	1.92 ± 0.54	0.84 ± 0.79	0.78 ± 0.02	15.70	0.00
10.81	2-Methyl-2-propenoic acid	2.56 ± 0.34	5.24 ± 0.97	2.18 ± 2.18	2.24 ± 0.21	18.09	0.00
11.55	Linalool oxide (pyranoid)	1.1 ± 0.11	2.41 ± 0.34	0.95 ± 1.03	1.07 ± 0.05	17.89	0.00
12.08	Benzoic acid, 2-hydroxy-, methyl ester	1.22 ± 0.11	0.99 ± 0.19	0.43 ± 0.4	0.92 ± 0.26	1.55	0.25
12.23	2-Cyano-2-methyl-1-cyclobutanol	0.93 ± 0.1	1.82 ± 0.42	0.78 ± 0.75	—	29.29	0.00
12.24	Artemisyl ketone	3.32 ± 1.93	6.57 ± 0.83	3.11 ± 2.49	2.34 ± 0.22	13.25	0.00
12.39	Elsholtzia ketone	7.92 ± 1.04	3.12 ± 0.72	1.63 ± 1.06	42.08 ± 2.33	564.41	0.00
13.84	Piperitone	4.74 ± 0.25	12.6 ± 2.44	5.1 ± 5.38	4.33 ± 0.19	26.57	0.00
15.35	β-Dehydroelsholtzia ketone	101.9 ± 5.79	107.73 ± 18.27	43.93 ± 45.4	50.38 ± 2.92	13.90	0.00
15.91	4-Acetyl-1-carbamoyl-5-methylpyrazole	18.16 ± 0.99	21.76 ± 2.95	8.57 ± 9.36	7.91 ± 0.6	28.80	0.00
17.67	β-Bourbonene	0.72 ± 0.42	1.15 ± 0.3	0.62 ± 0.38	0.73 ± 0.07	0.82	0.51
17.69	Dimethyl – heptadienone	3.61 ± 0.27	6.8 ± 0.91	2.66 ± 2.94	1.75 ± 0.17	36.18	0.00
18.47	Methyl 5-hydroxyiminopentanoate	1.1 ± 0.08	—	0.03 ± 0.04	2.93 ± 0.26	313.16	0.00
18.61	Caryophyllene	15.09 ± 0.95	14.86 ± 2.49	6.1 ± 6.23	25.05 ± 2.08	16.68	0.00
18.88	Hepta-2,4-dienoic acid, methyl ester	1.03 ± 0.08	1.55 ± 0.25	0.63 ± 0.66	—	52.42	0.00
19.56	α-Humulene	3.11 ± 0.21	2.79 ± 0.42	1.14 ± 1.17	4 ± 0.38	6.73	0.01
20.63	Bicyclogermacrene	—	0.28 ± 0.49	0.26 ± 0.2	0.85 ± 0.1	2.71	0.09
21.84	1-(2-Methoxyethenyl)cyclopropane	58.78 ± 2.18	86.98 ± 10.21	33.12 ± 38.22	28.58 ± 2.63	51.04	0.00
22.62	(+) Spathulenol	3.31 ± 0.21	6.49 ± 0.76	2.49 ± 2.84	2.87 ± 0.35	43.35	0.00
22.71	(−)-Caryophyllene oxide	4.76 ± 0.2	7.33 ± 0.88	2.8 ± 3.21	3.19 ± 0.33	43.20	0.00
23.19	2-[2-H3]Methylpyrazine	2.35 ± 0.2	3.69 ± 0.5	1.46 ± 1.58	0.9 ± 0.13	60.23	0.00
23.38	(−)-Humulene epoxide II	1.04 ± 0.05	1.4 ± 0.19	0.55 ± 0.61	2.07 ± 0.19	17.98	0.00
23.96	Humulenol-II	2.41 ± 1.43	3.72 ± 0.68	1.94 ± 1.29	0.81 ± 0.5	3.17	0.06
24.03	11,11-Dimethyl-4,8-dimethylenebicyclo[7.2.0]undecan-3-ol	1.33 ± 0.09	2.47 ± 0.28	0.95 ± 1.08	0.67 ± 0.47	17.32	0.00
24.48	Isoaromadendrene epoxide	4.43 ± 0.27	6.64 ± 0.84	2.58 ± 2.88	2.13 ± 0.23	45.55	0.00
24.82	(1R,7S,E)-7-Isopropyl-4,10-dimethylenecyclodec-5-enol	5.37 ± 0.26	8.52 ± 0.95	3.24 ± 3.74	2.1 ± 0.23	77.36	0.00
24.95	4-Isopropenyl-4,7-dimethyl-1-oxaspiro[2.5]octane	8.81 ± 0.42	8.02 ± 0.91	3.12 ± 3.47	5.7 ± 0.52	13.99	0.00
25.14	6-Heptyl-2,3,4,5-tetrahydropyridine	1.21 ± 0.1	2.2 ± 0.41	0.9 ± 0.93	—	48.81	0.00
25.88	1,1,4,7-Tetramethyldecahydro-1H-cyclopropa[e]azulene-4,7-diol	1.71 ± 0.11	3.46 ± 0.44	1.34 ± 1.51	1.59 ± 0.17	37.71	0.00
26.21	Nerolidol-epoxyacetate	7.67 ± 0.13	5.28 ± 0.64	2.02 ± 2.32	4.7 ± 2.14	3.65	0.04
26.26	Isospathulenol	0.25 ± 0.25	0.88 ± 0.22	0.45 ± 0.3	1.7 ± 2.62	0.61	0.62
26.47	Cholestan-3-ol, 2-methylene-, (3β,5α)-	1.98 ± 0.13	1.64 ± 0.21	0.66 ± 0.69	1.07 ± 0.15	14.24	0.00
26.79	(1R,2S,4S,5R,7R)-5-Isopropyl-1-methyl-3,8-dioxatricyclo[5.1.0.02,4]octane	1.31 ± 0.14	1.07 ± 0.19	0.47 ± 0.43	—	32.68	0.00
26.99	((4-Methoxyphenyl)dimethylacetyl)pyrrolidine	—	1.27 ± 0.52	0.6 ± 0.52	—	21.51	0.00
27.1	(−)-Isolongifolol, methyl ether	2.61 ± 0.3	2.47 ± 0.08	0.95 ± 1.08	1.17 ± 0.14	24.26	0.00
27.66	*cis-Z*-α-bisabolene epoxide	1.59 ± 0.14	1.85 ± 0.36	0.78 ± 0.76	1.09 ± 0.14	6.20	0.01
27.84	Diepicedrene-1-oxide	12.37 ± 0.68	11.98 ± 1.29	4.65 ± 5.19	9.95 ± 0.91	4.48	0.02
28.44	α-Kessyl acetate	1.2 ± 0.13	0.8 ± 0.15	0.36 ± 0.31	0.75 ± 0.44	2.16	0.15
28.63	6,10,14-Trimethyl-2-Pentadecanone	2.84 ± 0.14	1.94 ± 0.3	0.79 ± 0.81	1.13 ± 0.34	23.32	0.00
29.18	4,4,8-Trimethyltricyclo[6.3.1.0(1,5)]dodecane-2,9-diol	9.68 ± 0.56	10.73 ± 1.3	4.2 ± 4.63	7.21 ± 0.69	11.97	0.00
30.85	3-Methyl-2-butenoic acid, cyclobutyl ester	0.4 ± 0.23	1.16 ± 0.28	0.56 ± 0.43	—	22.58	0.00
31.11	Hexadecanoic acid	17.75 ± 1.33	13.37 ± 1.87	5.52 ± 5.55	6.04 ± 0.93	28.71	0.00
32.93	Oxalic acid, cyclohexyl decyl ester	1.69 ± 0.6	1.19 ± 0.05	0.61 ± 0.47	—	16.62	0.00
33.31	Carbonic acid, eicosyl vinyl ester	—	0.13 ± 0.22	0.12 ± 0.09	0.76 ± 0.46	4.03	0.03
33.46	*N*-Methyl-3-azabicyclo[3.1.0]hex-1-ylamine	—	0.2 ± 0.34	0.18 ± 0.14	—	4.24	0.03
34.12	(*Z*)-18-Octadec-9-enolide	6.32 ± 0.63	2.97 ± 1.87	1.82 ± 0.96	1.53 ± 0.46	10.89	0.00
34.23	9,12,15-Octadecatrienoic acid, (*Z*,*Z*,*Z*)-	10.37 ± 0.9	4.19 ± 0.87	1.99 ± 1.56	2.88 ± 1.06	36.47	0.00
34.75	Octadecanoic acid	2.74 ± 0.3	1.66 ± 0.34	0.77 ± 0.63	0.68 ± 0.08	24.98	0.00
34.87	Tetradecanamide	1.08 ± 0.27	1.19 ± 0.36	0.61 ± 0.41	2.84 ± 2.46	1.23	0.34
34.93	Hexadecanamide	4.07 ± 0.83	4.17 ± 0.99	2 ± 1.54	3.56 ± 2.19	0.49	0.70
35.53	Andrographolide	0.17 ± 0.29	1.05 ± 0.23	0.52 ± 0.37	—	22.60	0.00
37.81	Ethyl linoleate	2.2 ± 0.84	2.6 ± 0.96	1.47 ± 0.8	3.63 ± 1.17	1.24	0.34
37.93	9-Octadecenamide	33.31 ± 7.63	34.99 ± 8.27	16.96 ± 12.75	47.42 ± 11.1	1.40	0.29
39.29	Thunbergol	1.31 ± 0.09	1.81 ± 0.34	0.75 ± 0.76	0.84 ± 0.11	16.51	0.00
39.71	2-Methylhexacosane	3.89 ± 0.49	1.13 ± 0.11	0.58 ± 0.42	0.98 ± 0.15	65.93	0.00
40.29	Docosane	1.57 ± 2.72	2.98 ± 1.82	2.51 ± 0.5	2.51 ± 1.48	0.45	0.72
40.3	Triacontane	0.67 ± 0.39	—	0.13 ± 0.18	—	8.89	0.00
40.31	Heptacosane	7.83 ± 0.74	5.9 ± 0.87	2.5 ± 2.4	6.5 ± 2.43	1.13	0.38
41.39	3-Ethyl-tetracosane	0.79 ± 0.07	—	0.02 ± 0.03	—	368.24	0.00
41.65	2-Methyl-eicosane	1.36 ± 0.09	—	0.03 ± 0.04	—	623.81	0.00
42.27	Henicosanal	0.35 ± 0.6	0.9 ± 0.55	0.68 ± 0.15	—	2.33	0.13
42.72	11-Decyl-tetracosane	3.71 ± 0.29	1.46 ± 0.9	0.88 ± 0.48	1.24 ± 0.16	16.08	0.00
42.74	11-Decyl-docosane	—	—	—	1.74 ± 0.27	7.67	0.00
43.28	Celidoniol, deoxy-	2.68 ± 0.15	1.54 ± 0.31	0.67 ± 0.62	2.99 ± 0.32	16.81	0.00
43.28	Pentacosane	7.84 ± 0.5	4.37 ± 0.68	1.85 ± 1.78	6.31 ± 0.89	14.71	0.00
43.55	β-Sitosterol	5.06 ± 1.04	2.75 ± 2.46	2.08 ± 0.75	0.97 ± 1.04	3.32	0.06
43.89	Hentriacontane	39.09 ± 19.15	29.63 ± 17.41	22.06 ± 5.4	40.13 ± 23.5	0.17	0.91
44.22	13-Docosenamide, (*Z*)-	29.01 ± 1.19	31.4 ± 5.96	12.85 ± 13.26	33.39 ± 1.51	0.98	0.43
44.79	Squalene	2.71 ± 0.47	—	0.16 ± 0.22	—	100.12	0.00
45.78	2-Methyl-octacosane	5.77 ± 0.37	2.97 ± 0.59	1.31 ± 1.18	4.41 ± 0.96	9.01	1.0

a The data are expressed as the mean ± the standard deviation.

b "—" Not detected.

Under the high significance of difference level (*F* > 50, *P* < 0.05), the *E. rugulosa* pharmaceutical components with the highest relative content of the four origins were Elsholtzia ketone in Fumin, (1R,7S,E)-7-isopropyl-4,10-dimethylenecyclodec-5-enol in Dali, squalene in Lijiang, and these compounds can be labeled as origin characteristic medicinal components. However, in this condition, Yongsheng *E. rugulosa* has no pharmaceutical components whose content is significantly bigger than that of other origins.

### 3.2 PCA and HCA analysis

**Fig. 3:**
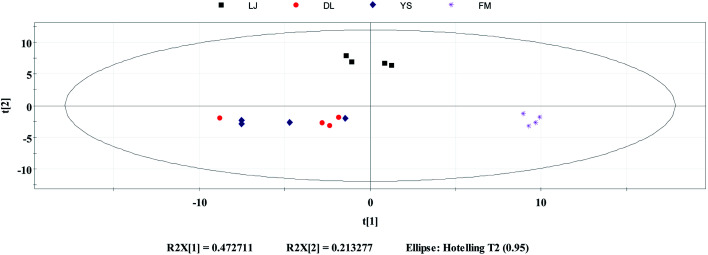
Principal component analysis: distribution of *E. rugulosa* samples from 4 origins in Yunnan Province.

PCA is an unsupervised classification method, which can objectively and directly reflect the classification of samples.^37^ It is commonly used as a reduction analysis method in multi-statistical analysis. PCA was performed for 4 different geo-origins of *E. rugulosa* using the Simca, and the results are shown in Fig. 3. As shown in Fig. 3, PCA first primary component variance interpretation rate is 47.2%, and the second primary component is 21.3%. Its total variance interpretation rate was 78.5%. As seen in the PCA plots, Fumin and Lijiang can be well distinguished when clustered together. However, Dali and Yongsheng can be partially distinguished, and the *E. rugulosa* of the two origins are very close.

It shows that the differences in volatile components of Fumin and Lijiang *E. rugulosa* can be clearly distinguished by PCA, while the difference of Dali and Yongsheng components is small. It may be due to their geographical and climatic characteristics. Fumin has a typical subtropical mountain monsoon climate with the lowest altitude, while Lijiang has a low-latitude warm temperate plateau monsoon climate with the highest altitude, so these two origins of *E. rugulosa* have obvious characteristics, respectively.^25,26^ Dali and Yongsheng belong to the transition from the subtropical mountain monsoon climate to the low-latitude warm temperate mountain monsoon climate, and the altitudes are relatively close.^25,26^ Therefore, the *E. rugulosa* characteristics of the two regions are relatively similar, and the difference is not obvious.

In terms of administrative division, Lijiang county and Yongsheng county are under the jurisdiction of Lijiang Autonomous Prefecture, while Dali county is under the jurisdiction of Dali Autonomous Prefecture. At the same time, Yongsheng *E. rugulosa* has similar characteristics to Dali, but is different from Lijiang county, indicating that the plants' characteristics are mainly influenced by geographical and climatic rather than administrative divisions.

The chemical composition of plant, indeed, is partially related to geographical and climatic conditions that are different among different geographical areas.^24,38,39^ Geographical and climatic conditions affect plants directly *via* the regulation of their biosynthetic pathways or indirectly *via* their effects on vine physiology and phenology.^38^ Ahmed found that the seasonality, water, geography, light factors, altitude, and temperature can cause 50% variation in secondary metabolites.^39^ Rienth research on grapes showed that temperature, water, and solar radiation govern the synthesis and degradation of primary (sugars, amino acids, organic acids, *etc.*) and secondary (phenolic and volatile flavor compounds and their precursors) metabolites.^38^ In the future, the influence of various geographical and climatic conditions on the metabolites of *E. rugulosa* will be systematically studied.

**Fig. 4:**
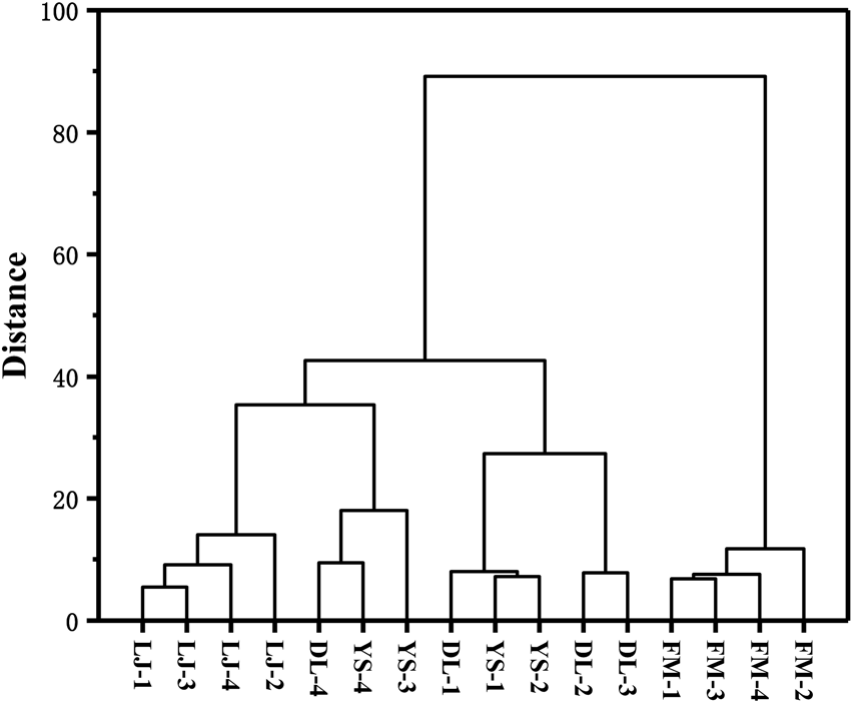
Hierarchical cluster analysis of the volatile compounds of *E. rugulosa* in Yunnan Province.

HCA is also an unsupervised objective classification method.^37^ It is a method of classifying samples according to the degree of similarity, which reflects the implicit similarity between samples. It combines the most similar samples together according to the degree of similarity between observations or variables and clusters the samples in a successive aggregation manner until all samples are finally clustered into one class. Generally, the smaller the critical value, the more similar the samples are.^37^ HCA analysis was carried out using OriginPro 2017 for *E. rugulosa* of 4 different origins. As shown in Fig. 3, when the critical value is between 45.2 and 89.3, Fumin *E. rugulosa* can be distinguished from the other three types of yebazi. When the critical value is between 14.3 and 18.4, Lijiang *E. rugulosa* can be distinguished. When the critical value is between 18.4 and 45.2, Dali and Yongsheng *E. rugulosa* can be divided into two types, but there is a certain intersection. Therefore, the two groups of Fumin and Lijiang *E. rugulosa* were well separated and clustered into two categories, respectively. In contrast, the Dali and Yongsheng *E. rugulosa* were poorly separated and clustered into three categories with a certain crossover. The results were consistent with the PCA results. In addition, it can be seen from Fig. 4 that the distance between Fumin and the other three origins is the largest, and the difference is the most obvious. The Lijiang samples are closer to the samples of Dali and Yongsheng, and the samples are more similar.

### 3.3 Correlation analysis

The correlation analysis of different components in *E. rugulosa* is of great significance for studying the correlation, metabolic pathway, and metabolic network among *E. rugulosa* metabolites. It can be used to understand the influence of geographical and climatic conditions on metabolites and the interaction process of different metabolites.

**Fig. 5:**
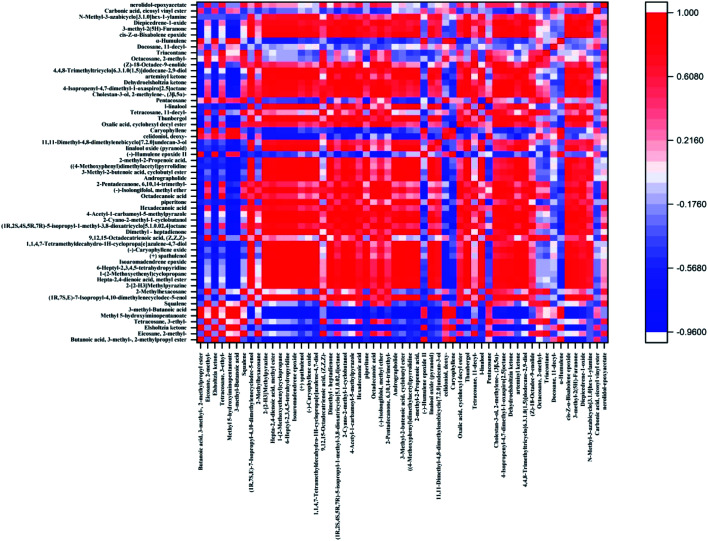
Heatmap of the differentially volatile compounds of *E. rugulosa* in Yunnan Province.

In this paper, the Pearson correlation coefficient between the compounds with a significance level of *P* < 0.05 was calculated, and the results are shown in Fig. 5. The absolute value of the correlation coefficient is limited to be above 0.85, and the *p* < 0.05, as the significant correlation condition. Under this condition, a total of 57 compounds, composed of 1596 pairs of compounds, of which 341 compound pairs with significant correlation were found, 300 pairs were positively correlated, and 41 pairs were negatively correlated (Fig. 5). In general, the positively correlated metabolite pairs have similar chemical composition, biological function, and homogeneous characteristics. Among them, the characteristic components of *E. rugulosa* Elsholtzia ketone are positively correlated with 2-methylpropyl 3-methylbutanoate, methyl 5-hydroxyiminopentanoate, 3-methyl-butanoic acid, caryophyllene, and negatively correlated with (1R,7S,E)-7-isopropyl-4,10-dimethylenecyclodec-5-enol, isoaromadendrene epoxide, (−)-isolongifolol, beta-dehydroelsholtzia ketone and so on. Elsholtzia ketone and 3-methyl-butanoic acid (hemiterpene derivatives), 3-methyl-butanoic acid (hemiterpene derivatives), caryophyllene (sesquiterpene) are all terpenoid metabolites and positively affected by terpenoid biosynthesis.^16^ Dehydroelsholtzia ketone may be obtained by dehydrogenation of Elsholtzia ketone, so they are negatively correlated. Therefore, Elsholtzia ketone is regulated by terpenoid metabolism. The results show that different geographical conditions have a great impact on terpenoid metabolic pathways.

### 3.4 *E. rugulosa* origin traceability functions and verification

From the above analysis, it can be seen that the volatile compounds of *E. rugulosa* from different origins have certain characteristics. Fisher discriminant is established based on the idea of variance.^40^ The principle is to minimize the variance between groups and maximize the variance between groups. Fisher discrimination is the most commonly used classic discrimination method. Compared with “black box” discrimination such as neural network and fuzzy mathematics, *etc.*, Fisher discrimination is “white box” discrimination. It can give the most influential components and their relative weights.^40^ Therefore, it is often used in the discriminant analysis of samples.

To further trace the origin of *E. rugulosa* from different origins, we constructed the origin traceability functions using the Fisher discriminant analysis. The GC-MS data were imported into the SPSS software, followed by step-by-step analysis and build the Fisher discrimination functions. Final screening of nine compounds as the representative function variable components are shown in Table 2.

**Table 2:** Fisher discrimination functions of 4 origins in Yunnan Province

Compounds	Geo-Origin			
	DL	FM	LJ	YS
2-Methylpropyl 3-methylbutanoate	−10110.886	740 116.143	74 993.793	−26440.045
Artemisyl ketone	130.395	−8997.888	−939.531	334.505
Methyl 5-hydroxyiminopentanoate	1851.893	−139925.356	−14677.245	4740.107
Humulenol-II	−75.955	4919.775	441.177	−206.877
Isospathulenol	−65.962	4431.237	429.954	−165.276
2-Pentadecanone, 6,10,14-trimethyl-	−293.419	23 916.656	2536.984	−828.038
Carbonic acid, eicosyl vinyl ester	−125.041	8120.202	682.405	−339.309
Eicosane, 2-methyl-	−2480.404	178 451.104	19 678.870	−6246.729
Octacosane, 2-methyl-	121.254	−7925.902	−707.265	337.847
Constants	−147.078	−474916.482	−5953.955	−787.601

The results of the functions are shown in Table 2. The four discriminant functions formed by these 9 indicators have variances of 82.0%, 13.5%, 3.4%, and 1.1% of the total variance, respectively, and the cumulative variance explanation rate is 100%. The resulting Fisher discriminant functions were the *E. rugulosa* origin discriminant function of 4 origins. In practical application, it is only necessary to substitute the contents of the above 9 indicators detected in the blind sample into the above functions, and the largest function value is the origin of the sample.

The validity of the model was verified by initial validation and LOOCV, and the results are shown in Table 3. In the initial verification results, all *E. rugulosa* from the four origins were correctly classified, and the initial validation accuracy was 100%. The initial verification training set and the verification set are the same set, and its results have a certain bias, and the accuracy rate is relatively high. Therefore, it is necessary to verify it with LOOCV.^41,42^

**Table 3:** The validation of *E. rugulosa* origin traceability functions

Method	Statistics	Geo-Origin	Predicted Geo-Origin				
			DL	LJ	YS	FM	total
Initial verification^a^	Number	DL	4	0	0	0	4
		LJ	0	4	0	0	4
		YS	0	0	4	0	4
		FM	0	0	0	0	4
	Accuracy%	DL	100	0	0	0	100
		LJ	0	100	0	0	100
		YS	0	0	100	0	100
		FM	0	0	0	100	100
LOOCV^b^	Number	DL	3	0	1	0	4
		LJ	0	4	0	0	4
		YS	1	0	3	0	4
		FM	0	0	0	4	4
	Accuracy%	DL	75	0	25	0	100
		LJ	0	100	0	0	100
		YS	25	0	75	0	100
		FM	0	0	0	100	100

a The total initial determination accuracy is 100%.

b The total LOOCV accuracy rate is 87.5%.

Leave-one-out cross-validation (LOOCV) is a special case of cross-validation. In each validation, nearly all the data except for a single observation are used for training, and the model is tested on that single observation. An accuracy estimate obtained using LOOCV is known to be almost unbiased. It is widely used when the available data are very rare.^41,42^ In the LOOCV results as shown in Table 3, the *E. rugulosa* of Lijiang and Fumin were all correctly classified, and the LOOCV accuracy was 100%; while the *E. rugulosa* of Dali, three were classified as Dali and one was classified as Yongsheng, and the LOOCV accuracy was 75%; *E. rugulosa* of Yongsheng, three classifications are Yongsheng, one is classified as Dali, and the LOOCV accuracy was 75%; the overall leave-one-out cross-validation accuracy rate of the model was 87.5%.

The analysis results of LOOCV were consistent with the results of PCA and HCA, which may be caused by the similarity of the volatile components of Yongsheng and Dali *E. rugulosa*. It indicates that the GC-MS combined with multivariate statistical analysis for origin traceability has certain limitations, namely, when the geographical climate of the origin is similar and then result in the difference of volatile herbal volatile components may not be obvious, the classification is prone to misjudgment. However, herbal medicines with insignificant differences in chemical composition from different origins also have small differences in efficacy. Therefore, this misjudgment of origin has a limited impact on the efficacy and use of Chinese herbal medicines.

To sum up, the *E. rugulosa* origin traceability based on Fisher's stepwise discriminant analysis, the initial verification accuracy was 100%, and LOOCV accuracy was 87.5%, which can realize the identification of most *E. rugulosa* origins.

## 4. Conclusions

In this study, volatile components were determined in 16 *E. rugulosa* flower samples from 4 regions including Lijiang, Dali, Yongsheng, and Fumin. The GC-MS data were analyzed using ANOVA. The results showed that more than 17 active ingredients were identified in *E. rugulosa*. The origin characteristic medicinal components of *E. rugulosa* are Elsholtzia ketone in Fumin, (1R, 7S, e)-7-isopropyl-4,10-dimethylenecyclodec-5-enol in Dali, squalene in Lijiang, respectively, while Yongsheng has no obvious high medicinal components. PCA and HCA analysis show that the *E. rugulosa* samples of Fumin and Lijiang can be clearly classified, Dali and Yongsheng *E. rugulosa* can be partially distinguished but there is a certain overlap. This is due to the large difference in geography and climate between Fumin and Lijiang, while Dali and Yongsheng belong to the middle of the geographical climate transition zone with a certain crossover. In addition, the Pearson correlation coefficient was used to study the correlation of the chemical components of the *E. rugulosa* components. The results showed that *E. rugulosa* characteristic component Elsholtzia ketone is regulated by terpenoid metabolism. The effective identification of *E. rugulosa* origin is achieved by Fisher step-by-step discriminant analysis, and the initial verification and LOOCV accuracy of the *E. rugulosa* origin traceability functions are 100% and 87.5%, respectively.

The GC-MS combined with multivariate statistical analysis can not only determine the volatile components of Chinese herbal medicines, but also realize their origin traceability, which can be widely used in the efficacy evaluation and origin traceability. The *E. rugulosa* volatile compounds are rich in active antiviral ingredients, which may have efficacy in preventing, anti- or/and ease COVID-19, and it will be further studied in the future.

## Conflicts of interest

There are no conflicts to declare.

## Supplementary Material
